# lncRNA profile of *Apis mellifera* and its possible role in behavioural transition from nurses to foragers

**DOI:** 10.1186/s12864-019-5664-7

**Published:** 2019-05-21

**Authors:** Fang Liu, Tengfei Shi, Lei Qi, Xin Su, Deqian Wang, Jie Dong, Zachary Y. Huang

**Affiliations:** 1 College of Animal Science and Technology, Anhui Agricultural University, Hefei, 230000 Anhui China; 2 Institute of Animal Husbandry and Veterinary Medicine, Zhejiang Academy of Agricultural Sciences, Zhejiang, 310021 Hangzhou China; 3 Department of Entomology, Michigan State University, East Lansing, MI 48824 USA

**Keywords:** lncRNA, mRNA, Behavioural transition, Foragers, Nurses

## Abstract

**Background:**

The behavioural transition from nurses to foragers in honey bees is known to be affected by intrinsic and extrinsic factors, including colony demography, hormone levels, brain chemistry and structure, and gene expression in the brain. However, the molecular mechanism underlying this behavioural transition of honey bees is still obscure.

**Results:**

Through RNA sequencing, we performed a comprehensive analysis of lncRNAs and mRNAs in honey bee nurses and foragers. Nurses and foragers from both typical colonies and single-cohort colonies were used to prepare six libraries to generate 49 to 100 million clear reads per sample. We obtained 6863 novel lncRNAs, 1480 differentially expressed lncRNAs between nurses and foragers, and 9308 mRNAs. Consistent with previous studies, lncRNAs showed features distinct from mRNAs, such as shorter lengths, lower exon numbers, and lower expression levels compared to mRNAs. Bioinformatic analysis showed that differentially expressed genes were mostly involved in the regulation of sensory-related events, such as olfactory receptor activity and odorant binding, and enriched Wnt and FoxO signaling pathways. Moreover, we found that lncRNAs TCONS_00356023, TCONS_00357367, TCONS_00159909 and mRNAs *dop1*, *Kr-h1* and *HR38* may play important roles in behavioural transition in honey bees.

**Conclusion:**

This study characterized the expression profile of lncRNAs in nurses and foragers and provided a framework for further study of the role of lncRNAs in honey bee behavioural transition.

**Electronic supplementary material:**

The online version of this article (10.1186/s12864-019-5664-7) contains supplementary material, which is available to authorized users.

## Background

The Western honey bee, *Apis mellifera L.*, a social insect and a good agricultural pollinator, possesses a remarkable trait: the behavioural transition from inhive tasks to outside tasks. In a typical colony, young worker honey bees (nurses) take care of the brood; approximately one week later, worker bees change to new roles, such as storing and processing food in colonies; when they are three weeks of age, worker bees begin to forage for honey, pollen, propolis or water outside of the hive [1]. Foragers can change back to be nurses under certain conditions [2]. Such behavioural plasticity has attracted much research attention, and a number of factors have been reported to be associated with it: colony demography [3], hormone levels [4], exocrine gland activity [5], brain chemistry and structure [6], circadian rhythms [7], and gene expression in the brain [8]. Non-coding RNAs (ncRNA) are those RNAs not involved in coding; they include small RNAs (18–35 nucleotides, nt), and long non-coding RNAs (lncRNA), with lengths > 200 nt. lncRNAs can be classified as natural antisense transcripts, long intronic non-coding RNAs, or long intergenic non-coding RNAs (lincRNAs) according to their genomic position [9]. In recent years, lncRNAs have been confirmed to play roles in many biological processes, such as cell differentiation and development, immune responses and tumourigenesis [10–12]. A most recent study showed that most lncRNAs were dysregulated in a tumour-specific manner and synergistically dysregulated cancer pathways in multiple tumour contexts [13]. Many *Drosophila melanogaster* lncRNAs have been found to be associated with X inactivation [14, 15], behaviour [16, 17], and neuronal disease [18].

lncRNAs in honey bees have also been observed to function in developmental processes. Both *AncR-1* and *Ks-1* have abundant expression in the brain and accumulate in the nucleus, showing their potential role in the regulation of neural function in honey bees [19, 20]. *Kakusei* in the nucleus is activated in a subset of neurons in the brains of dancing foraging bees, and *Nb-1* is involved in regulating octopamine and juvenile hormone release during the behavioural transition from nursing to foraging [21, 22], suggesting that they are involved in the regulation of behaviours in honey bees. Jayakodi et al. (2015) identified lincRNAs specifically associated with viral diseases in honey bees, and they were preferentially expressed in ovary tissue [23]. Chen et al. (2017) further observed dramatic expression changes of coding and noncoding RNAs, suggesting that they play a critical role in oviposition in honey bee queens [24]. A context-dependent transcription of one lncRNA encoding an anti-sense transcript of lysosomal alpha-mannosidase in the honey bee has been shown to be linked to DNA methylation [25]. However, there are no reports about the role of lncRNA in the division of labour in honey bees.

In this study, we used RNA-seq to detect the profile of lncRNA in the heads of nurses and foragers from typical colonies and obtained many differentially expressed transcripts. In addition, to separate whether the nurse-forager difference was due to age or task performance, we compared lncRNA profiles of bees in typical colonies with those of bees from single-cohort colonies.

## Results

### lncRNAs identified from RNA-seq

**Fig. 1:**
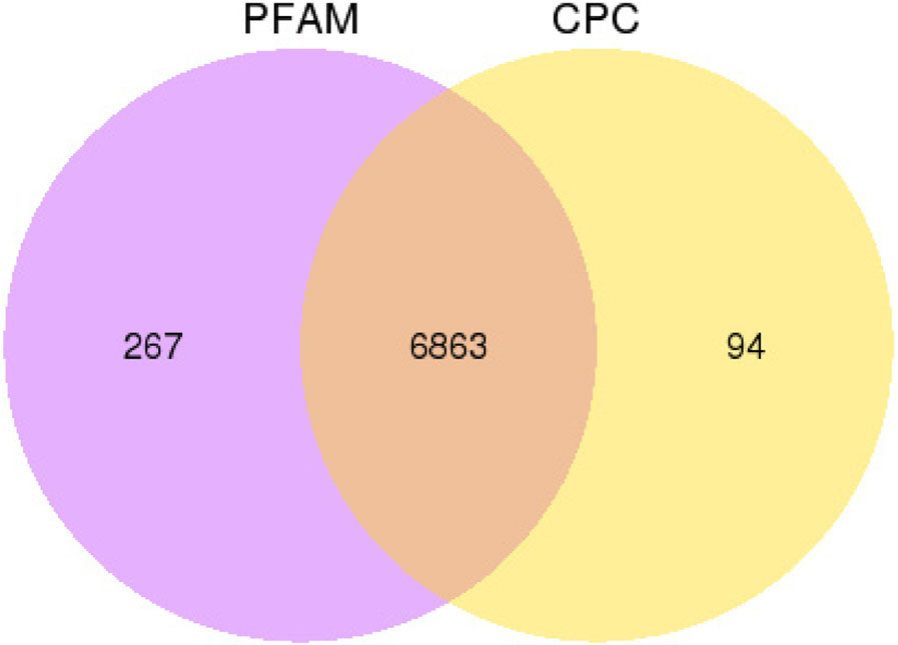
Identification of non-coding lncRNAs by using PFAM and CPC. A total of 6863 non-coding transcripts were selected using two software programs evaluating protein-coding transcripts to remove putative protein-coding transcripts

Total RNA from the heads of six honey bee groups (TC_N, TC_F, SCC_YN, SCC_YF, SCC_ON, SCC_OF, see methods for explanation) was isolated and sequenced. Approximately 50 to 102 million raw reads and 49 to 100 million clean reads per sample were obtained by RNA-Seq (Additional file 1). The sequence reads were mapped with a reference annotation. Approximately 82.51 to 86.79% of the reads were mapped to mRNAs, depending on the group (Additional file 2). For ncRNAs, 1.17 to 2.08% of the sample reads were mapped (Additional file 2). We identified 7470 novel lncRNAs after using Cufflinks (v2.1.1) [26] and Scripture (beta2) [27]. Then, 6863 putative non-coding transcripts were obtained using Coding Potential Calculator (CPC) and Pfam-scan (PFAM) (Fig. 1). We also obtained 22,203 transcripts of mRNAs, with 21,596 transcripts mapped to the reference genome, with the remaining coding transcripts (607) not annotated.

### Comparative features of mRNAs and lncRNAs

**Fig. 2:**
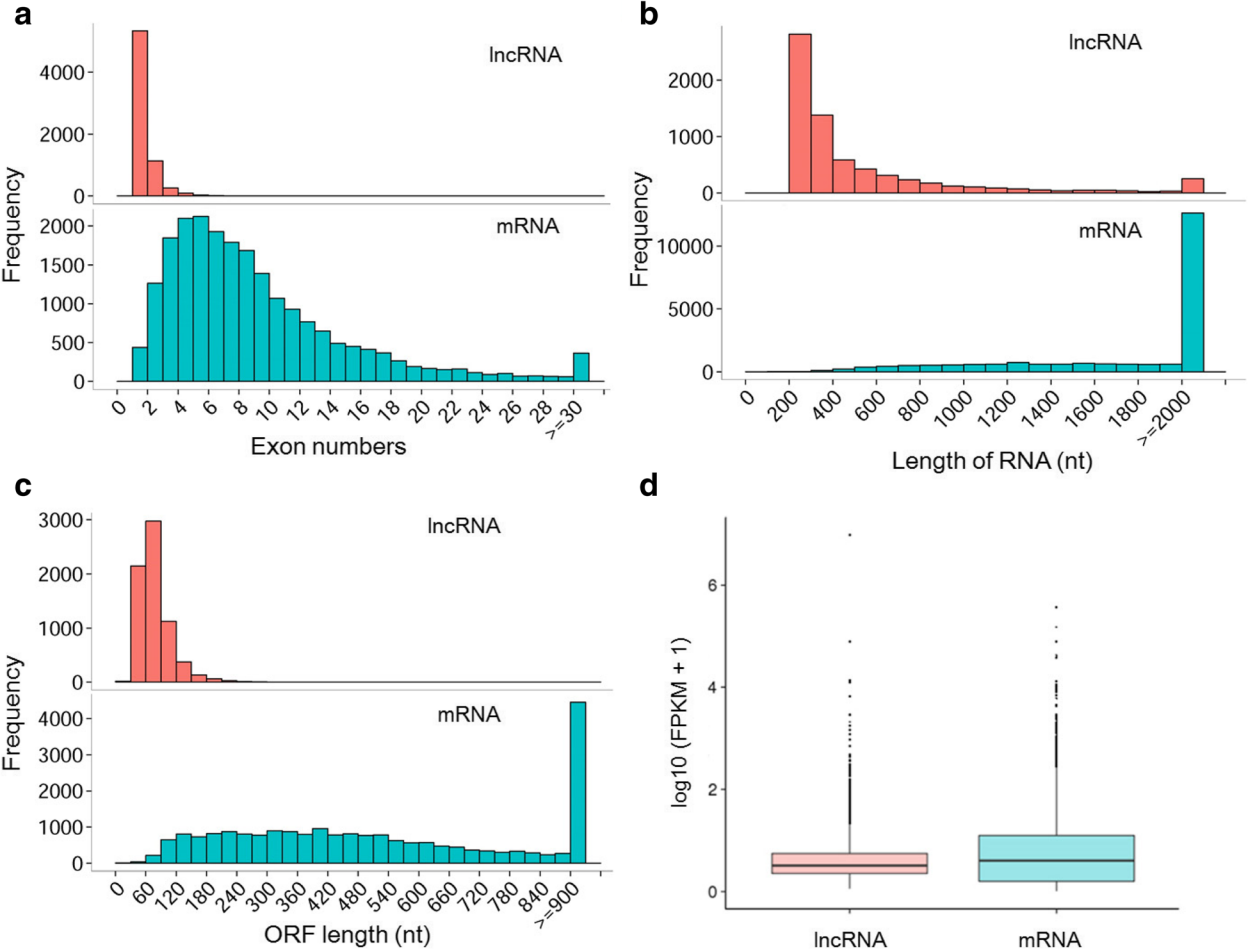
Exon number distribution of coding transcripts (mRNAs) and lncRNAs (**a**). Length distribution of 6863 new predicted lncRNAs (red) and 21,597 coding transcripts (blue) (**b**). ORF length distribution of mRNAs and lncRNAs (**c**). Expression level indicated by log10 (FPKM + 1) in the mRNAs and lncRNAs (**d**)

A total of 21,596 mRNAs and 6863 lncRNAs were obtained from the honey bee heads (all samples combined). Most lncRNAs contained one to two exons, which was significantly different from that of mRNAs (Chi-square test, df = 29, χ^2^ = 21,019.93, *P* < 0.0001, Fig. 2a), and there was a great divergence in the distribution of transcript length between mRNAs and lncRNAs (Chi-square test, df = 19, χ^2^ = 3701.49, P < 0.0001, Fig. 2b). Moreover, we found that most lncRNAs had significantly shorter ORFs ranging from 30 bp to 240 bp (Chi-square test, df = 30, χ^2^ = 22,413.34, P < 0.0001) compared to mRNAs (30 to > 900 bp) (Fig. 2c), and lncRNAs showed a significantly lower (T-test, P < 0.0001) expression level compared to mRNAs (Fig. 2d).

### Differentially expressed genes between nurses and foragers

**Fig. 3:**
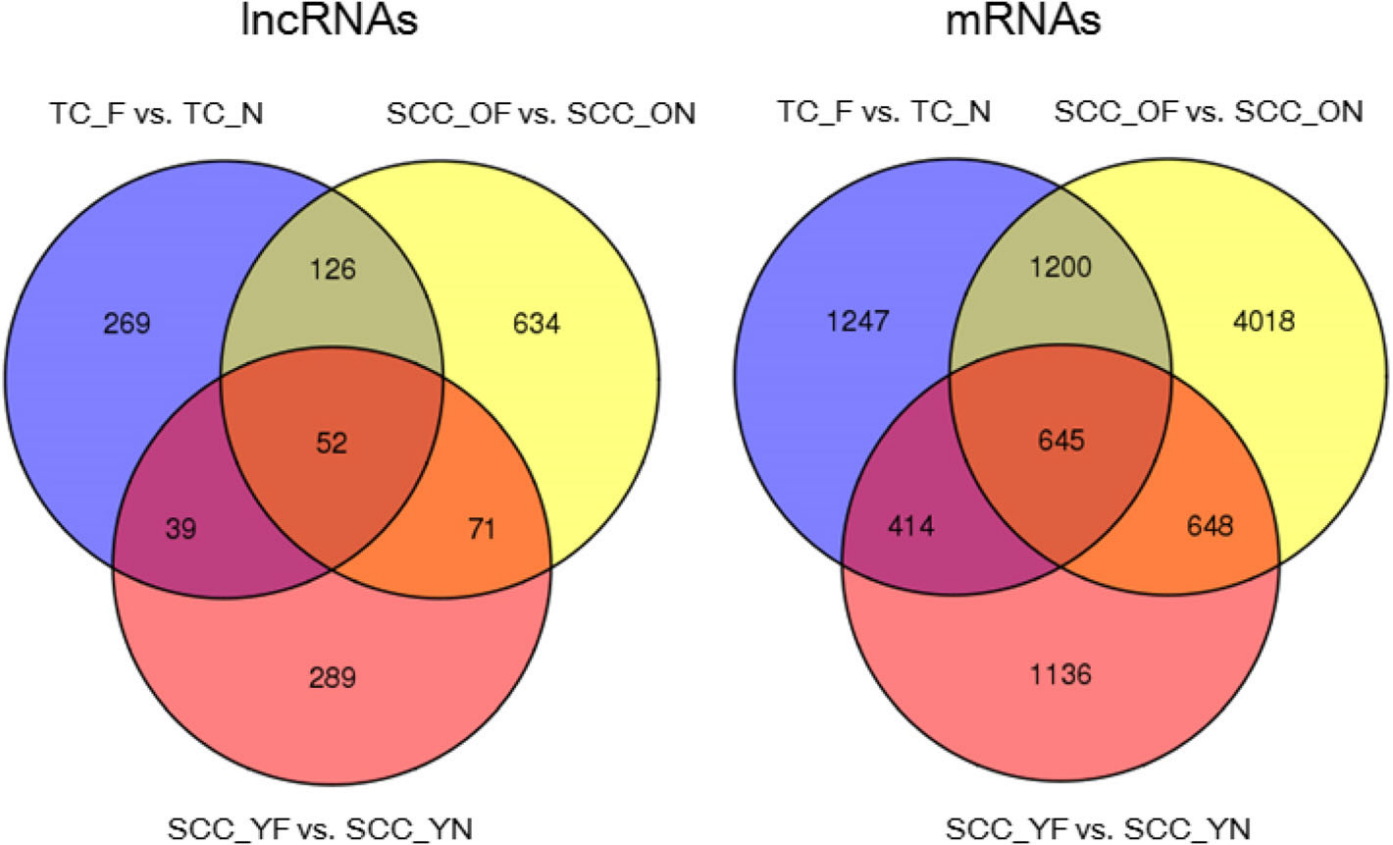
Gene expression profiles and number of differentially expressed genes for honey bees. Venn diagram of common differentially expressed genes (lncRNA and mRNA) among the three comparison groups

A total of 1480 differentially expressed lncRNAs and 9308 mRNAs were detected from pairwise nurse-forager comparisons in samples from typical colonies and single-cohort colonies using Cufflinks (v2.1.1) [26] (Table 1). The heatmaps of these genes are displayed in Additional file 3. A total of 449 upregulated and 37 downregulated lncRNAs were detected in the TC_F vs. TC_N comparison, 846 and 37 in the SCC_OF vs. SCC_ON comparison, and 368 and 83 in the SCC_YF vs. SCC_YN comparison, respectively. Additionally, 2925 upregulated and 581 downregulated mRNAs were detected in the TC_F vs. TC_N comparison, 6116 and 395 in the SCC_OF vs. SCC_ON comparison, and 2031 and 812 in the SCC_YF vs. SCC_YN comparison, respectively. As shown in Figs. 3, 52 differentially expressed lncRNAs and 645 differentially expressed mRNAs were common among the three contrasts (Additional file 4). We also found that 123 differentially expressed lncRNAs and 1293 differentially expressed mRNAs were common between the SCC_OF vs. SCC_ON and SCC_YF vs. SCC_YN comparisons (Additional file 5).

**Table 1:** Number of differentially expressed transcripts in each comparison

Transcripts		TC_F vs. TC_N	SCC_OF vs. SCC_ON	SCC_YF vs. SCC_YN
lncRNA	Up-regulated	449	846	368
	Down-regulated	37	37	83
mRNA	Up-regulated	2925	6116	2031
	Down-regulated	581	395	812

### GO and KEGG analysis of target genes

**Fig. 4:**
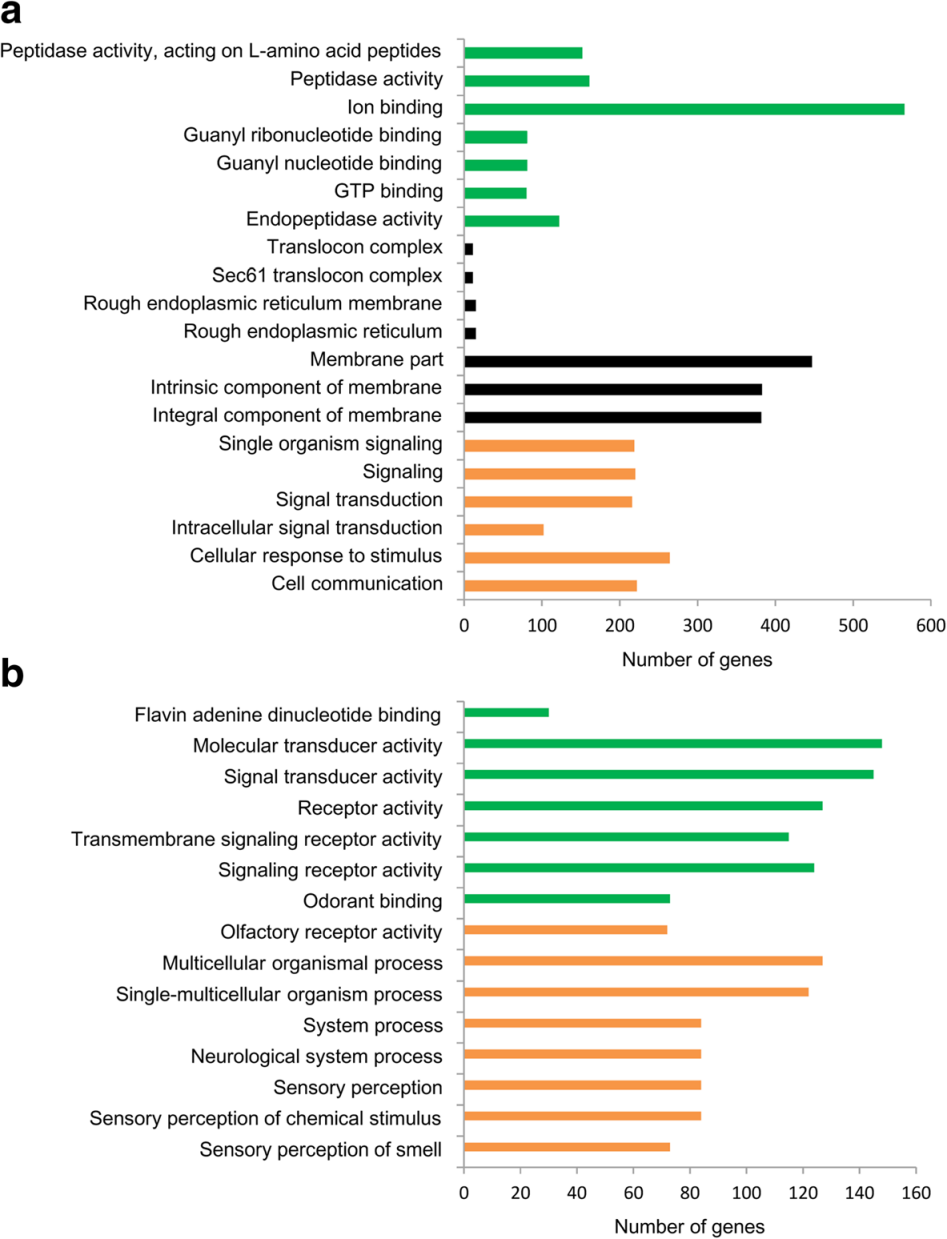
The significant enrichment GO terms (corrected *p* value < 0.05) detected in the TC_F vs. TC_N (**a**) and SCC_YF vs. SCC_YN (**b**) comparisons (*cis*). Green bars represent molecular function terms; black bars represent cellular component terms; orange bars represent biological process terms

We predicted the potential targets of lncRNAs by *cis* and *trans* regulation. A total of 3992 target genes (10 kb) and 9452 target genes (100 kb) were predicted in the *cis* role (data not shown). GO analysis showed that 20 significantly enriched GO terms (corrected *p*-value < 0.05) were detected in the TC_F vs. TC_N comparison and 15 terms were predicted in the SCC_YF vs. SCC_YN comparison, and most of the GO terms were related to regulation of sensory behaviour, such as sensory perception of smell, olfactory receptor activity and odorant binding (Fig. 4). However, there was no significant enrichment of GO terms for the SCC_OF vs. SCC_ON comparison. The most enriched pathways in the three comparisons of honey bees included “carbon metabolism”, “glycine, serine and threonine metabolism”, and the “phosphatidylinositol signalings system”. Among the top 20 enriched pathways, “Hippo signaling pathway - fly” and “Wnt signaling pathway” were the common pathways in the three comparison groups (Additional file 6).

For the *trans* action of lncRNAs, 10,196 target genes were predicted. There were 118 significantly enriched GO terms (corrected p-value < 0.05) between the TC_F vs. TC_N comparison, 110 terms in the SCC_YF vs. SCC_YN and 46 terms in the SCC_OF vs. SCC_ON comparison (Additional file 7). These GO terms included a variety of molecular functions. We also found that “binding” and “protein binding” were the most common significantly enriched terms in each of the honey bee comparisons. The most enriched pathways in the three comparisons (TC_F vs. TC_N, SCC_YF vs. SCC_YN, SCC_OF vs. SCC_ON) of honey bees were the “endocytosis”, “ubiquitin mediated proteolysis”, and “FoxO signaling pathway”, respectively. Among the top 20 enriched pathways, the “Wnt signaling pathway” was the most common pathway in all three comparisons (Additional file 8).

### Functional analysis of mRNA in honey bee samples

GO and KEGG enrichment analyses were also performed with differentially expressed mRNAs. In the TC_F vs. TC_N comparison, a total of 60, 24, and 62 GO terms were significantly enriched in the biological process, cell component, and molecular function, respectively. In the SCC_OF vs. SCC_ON comparison, 44, 11 and 45 GO terms were significantly enriched in the biological process, cell component, and molecular function, respectively. In the SCC_YF vs. SCC_YN comparison, 17, 14 and 37 were significantly enriched in the biological process, cell component, and molecular function, respectively (Additional file 9). The most enriched terms were associated with the gene expression and behaviour of honey bees. “Protein binding”, “binding”, “signaling”, “signal transduction”, “response to stimulus”, “Wnt signaling pathway”, “neuroactive ligand-receptor interaction”, “FoxO signaling pathway”, “notch signaling pathway”, and the “Hippo signaling pathway - fly” were common pathways among the top 20 enriched pathways in the three comparisons (Additional file 10). Interestingly, we observed that the “Wnt signaling pathway” was the most enriched pathway in all three comparisons.

### Validation of selected lncRNAs and mRNAs

**Fig. 5:**
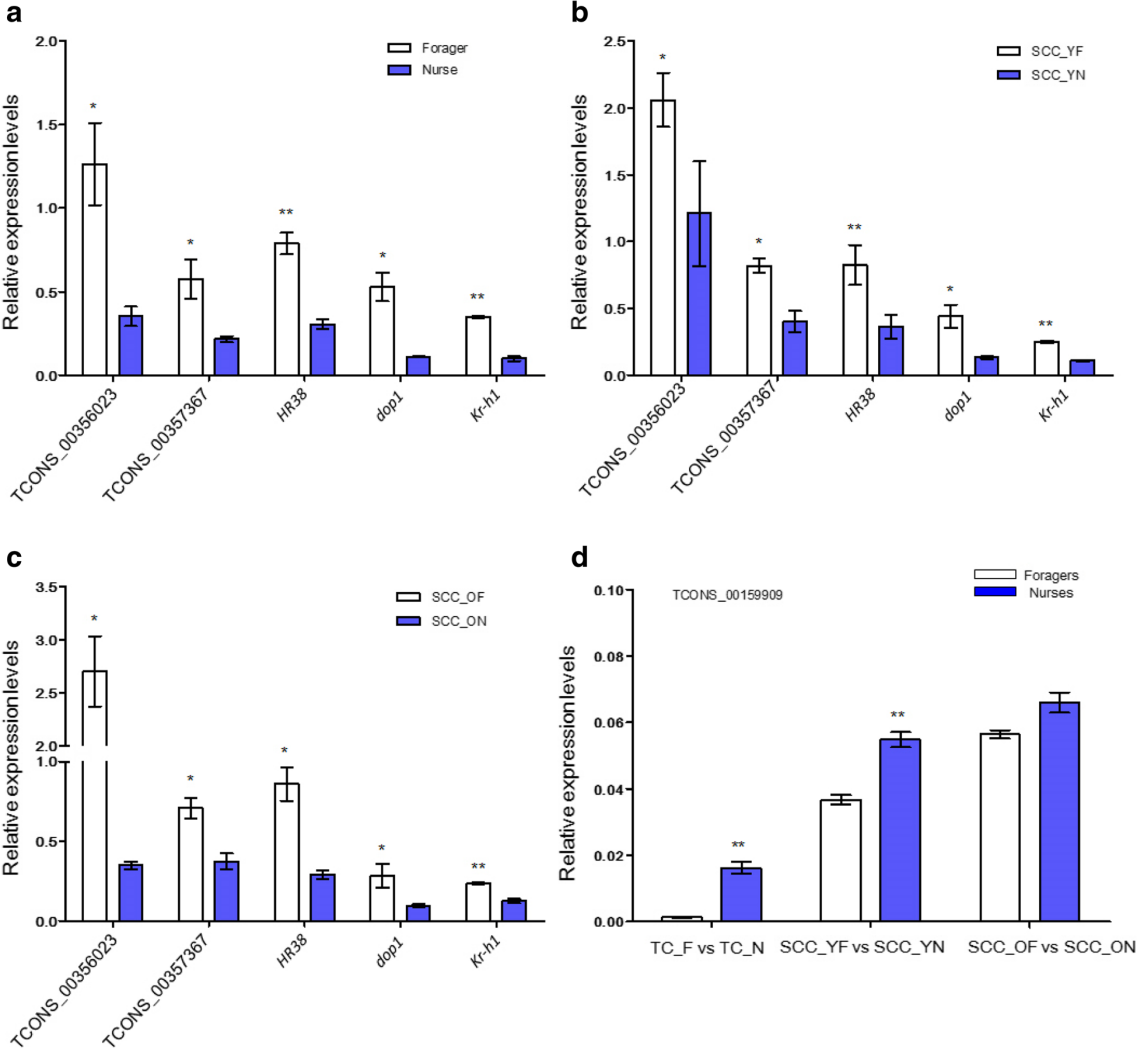
Validation of selected lncRNAs and mRNAs by using quantitative PCR. The relative expression levels of TCONS_00356023, TCONS_00357367, *HR38, dop1*, *Kr-h1* in the heads of nurses and foragers from typical colonies (**a**); the relative expression levels of TCONS_00356023, TCONS_00357367, *HR38, dop1*, *Kr-h1* in the heads of normal nurses and precocious foragers from single-cohort colonies (**b**); the relative expression levels of TCONS_00356023, TCONS_00357367, *HR38, dop1*, *Kr-h1* in the heads of overaged nurses and normal-aged foragers from single-cohort colonies (**c**); the relative expression level of TCONS_00159909 in the heads of nurses and foragers from both typical colonies and single-cohort colonies. TCONS_00159909 expression was presented in a single figure, as its expression level was too low to be shown in the same plot as the other two lncRNAs (**d**). Blue represents nurses, and white represents foragers. Data were normalized to the housekeeping gene *β-actin* and are shown as the mean ± standard error, *P < 0.05, **P < 0.01

To validate the RNA-seq results, we chose three lncRNAs (TCONS_00357367, TCONS_00356023, TCONS_00159909) and three mRNAs (*dop1, Kr-h1, HR38*) for quantitative PCR (qPCR) (Table 2). The validated lncRNAs were significantly differentially expressed among the comparison groups, and the predicted target genes of these lncRNAs were previously shown to be associated with the division of labour in honey bees. The mRNAs selected were significantly differentially expressed among the three honey bee comparison groups. As shown in Fig. 5, TCONS_00357367, TCONS_00356023, *dop1, Kr-h* and *HR38* had significantly higher expression in foragers than in nurses, which is consistent with our RNA-seq data. TCONS_00159909 had significantly higher expression in honey bee nurses than in foragers in the TC_F vs. TC_N and SCC_YF vs. SCC_YN comparisons but did not show significantly different expression between the SCC_ON and SCC_OF comparison (Fig. 5).

**Table 2:** The sequences of primers of the selected lncRNAs and mRNAs

Name of primers	Sequences(5’to3’)
TCONS_00356023-F	TTGAGACGACATTAAGACAGA
TCONS_00356023-R	CCACTGATTCTATTCCTTCCT
TCONS_00357367-F	TTATTCATCGGTGGATTA
TCONS_00357367-R	GTTCATCTCTTGTCTTAC
TCONS_00159909-F	GCGCCACCACGTTCGATCATC
TCONS_00159909-R	ACTCGGCTACGTGACCGTGAC
Kr-h1-F	GTAGAAGAGTCGAGGCTGCATTGG
Kr-h1-R	CACAGGATTGCTACTTGGAGGAGTTAG
dop1-F	ATCGCTGTAGTGTGGTTGCTC
dop1-R	GGATGTTCTTCTTTGCTATCGTC
Hr38-F	AGCCGACTGGTAATATCA
Hr38-R	TTCCTTCCTTCCTTCCTT
β-actin-F	TGCCAACACTGTCCTTTCTG
β-actin-R	AGAATTGACCCACCAATCCA

## Discussion

In this study, RNA-seq was performed to build the lncRNA and mRNA profiles of honey bees. We obtained 6863 putative non-coding transcripts, 1480 differentially expressed lncRNAs, and 9308 differentially expressed mRNAs in the nurse vs. forager comparisons in bees from both typical colonies and single-cohort colonies. A total of 52 significantly differentially expressed lncRNAs and 645 such mRNAs were shared among the three comparisons (TC_F vs. TC_N, SCC_OF vs. SCC_ON and SCC_YF vs. SCC_YN, Fig. 3). Bioinformatic analysis showed that the “Wnt signaling pathway” may play an important role in the behavioural changes of honey bees.

lncRNA have been involved in essential biological processes, such as imprinting, gene regulation and dosage compensation, especially in mammals [28]. It has been reported that lncRNA regulates sleep behaviour, locomotor behaviour and immunity in *Drosophila* [29–31]. In this study, we found that several lncRNAs were actively involved in honey bee behavioural changes. First, lncRNAs TCONS_00207749 and TCONS_00207751 had lower expression in foragers than in nurses, and they both target the *foraging gene* (a *cGMP-dependent protein kinase*) in *cis*. The gene *foraging* was shown to have significantly higher expression in foragers than in nurses and plays an important role in regulating the behavioural transition from nursing to foraging [32]. Because of their inhibitory role towards *foraging*, their lower expression levels in foragers are consistent with the higher expression of *foraging* in foragers. Therefore, these two lncRNAs may be involved in modulating honey bee behaviour via modulating f*oraging*. Furthermore, we found that lncRNA TCONS_00357367 was upregulated in foragers from both typical and single-cohort colonies (Additional file 4), while its target gene ACSF2 (acyl-CoA synthetase family member 2) in *cis* was downregulated in foragers in these colonies. Acyl-CoA synthetases (ACS) carry out a fundamental reaction in fatty acid metabolism [33], and dysregulation of fatty acid metabolism by disruption of ACS function in vivo can lead to neurodegenerative pathologies, which is evident in neuronal cells of *Drosophila* [34]. Foragers (typically older bees) had lower lipid amounts than did nurses (typically young bees) [35]. Here, we found that ACSF2 was significantly involved in the molecular function, catalytic activity, and metabolism processes. TCONS_00356023 was also upregulated in foragers in both typical and single-cohort colonies, and it targets Vg (vitellogenin) in *cis*. This is consistent with previous findings of lower Vg expression in foragers than in nurses [36] (Additional file 4). Early foraging onset can be induced by inhibition of vitellogenin mRNA (*vg*) production through RNAi [37]. Moreover, TCONS_00159909 was upregulated in nurses, and its predicted targets in *cis* and *trans* included major royal jelly proteins (*mrjp1-mrjp9*), *yellow-e*, *yellow-e3, yellow-g*, *yellow-g2* and *yellow-h*. The MRJP protein family is evolved from the ancient Yellow protein family [38]. *Mrjps* were found to be expressed in the mushroom bodies of the honey bee brain. *Mrjp1*, coding for the most abundant protein of larval food, may regulate the learning ability of the honey bee [39, 40]. The expression of TCONS_00357367, TCONS_00356023 and TCONS_00159909 were further confirmed by qPCR (Fig. 5), the results of which were consistent with the RNA-seq data.

Over 1500 genes are differentially expressed in the brains of nurses and foragers [41]. *Foraging* and *malvolio* are among the presumably many genes that play a causal role in the division of labour of honey bees [32, 42]. In the present study, we found that several mRNAs were actively involved in honey bee behavioural changes. AmDOP1 was shown to be highly abundant in the soma of mushroom body intrinsic neurons of honey bees and was involved in signal processing of visual and olfactory information [43]. It has similar function to the *Drosophila* DAMB (dopamine receptor in mushroom bodies) gene, which plays key roles in arousal and memory [44]. In this study, we found that *dop1* had a high expression in foragers but no expression in nurses in both typical and single-cohort colonies. We also found that *dop1* was significantly enriched in the “signal transduction”, “signaling”, and “response to stimulus” terms and was related to the “neuroactive ligand-receptor interaction” pathway, suggesting its importance in the function of honey bee behaviour. Expression of the transcription factor *Krüppel-homolog 1* (*Kr-h1*) was significantly higher in foragers than in nurses and is associated with cGMP-mediated changes in the brain that occur early in the transition to foraging behaviour [41]. Here, we found that *Kr-h1* was upregulated in foragers in both typical and single-cohort colonies and involved in “signal transduction”, “signaling”, “response to stimulus”, and “binding” terms. A recent study showed that *HR38* mediated 20-hydroxyecdysone regulating carbohydrate metabolism during mosquito reproduction [45]. In this study, we found that *HR38* was upregulated in foragers in both typical and single-cohort colonies, which is similar to the results of the Khamis et al. (2015) study, which showed that *HR38* had a higher expression in the brain of foragers compared to nurses and was concentrated in a subset of the mushroom body neurons of foragers [46]. The HR38-mediated pathway (ecdysteroid signaling) in the mushroom bodies was suspected to be involved in the division of labour by the workers [47]. Furthermore, the expression levels of *dop1*, *Kr-h1*, and *HR38* were confirmed by qPCR in this study (Fig. 5), and were consistent with the RNA-seq data. These results suggest that these genes may play important roles in the honey bee behavioural transition.

Through bioinformatic analysis, we found that the “Wnt signaling pathway” was the most enriched pathway both in mRNAs and the target genes of lncRNAs. This suggests that Wnt may play a critical role in honey bee behavioural maturation. The honey bee genome has the same number of *Wnt* genes as that of *Drosophila*, and many features of Wnt signaling are conserved between the two species [48]. Wnt signaling is involved in embryogenesis and imaginal disc development in *Drosophila* [49]. Additionally, it can cross-talk with JH (juvenile hormone)-signaling by suppressing transcription of genes encoding for putative JH receptors to induce downregulation of *Kr-h1*expression in the early larval stages of *Drosophila* [50]. JH plays a critical role in honey bee development, including the regulation of division of labour [51]. Honey bees lacking the hormone would perform foraging later than normal bees [52]. Wnt signaling is one of the most crucial morphogens for development; during the maturation of the central nervous system its action is associated with the establishment and maintenance of synaptic structure and neuronal function [53]. Dysregulated Wnt signaling can lead to disorders of behaviour [54]. In this study, we found that TCONS_00357367 also targets the gene *Pkc* (protein kinase C), which was enriched in the Wnt signaling pathway, and it had significantly higher expression in foragers than in nurses. PKC had high expression in mushroom bodies and the antennal lobes, neuropils involved in the processing of sensory information and in learning [55]. In *Drosophila* inhibited PKC leads to a dissociation of their acquisition of learning and memory from their performance of a task [56]. Taken together, we deduce that the Wnt signaling pathway may be involved in the modulation of honey bee behaviour by regulating the neuronal function of the honey bee brain or that it may interact with the JH pathway to affect honey bee behaviours.

## Conclusions

We first generated the expression profile of lncRNA in nurses and foragers by deep RNA-seq. Bioinformatic analysis showed that some lncRNAs and mRNAs were involved in important biological processes associated with honey bee behaviours, such as sensory perception of smell, olfactory receptor activity and odorant binding, and these lncRNAs may be involved in regulating the division of labour in honey bees by targeting mRNAs. Additionally, we found that “Wnt signaling pathway” may be involved in honey bee behavioural transition. As the research of lncRNAs just started a few years ago, and little was known about its function in honey bees, our study should provide important resources for studying lncRNAs with regard to the behavioural plasticity of honey bees.

## Methods

### Honey bee collections

Honey bees (*Apis mellifera*) were kept according to standard beekeeping practices at Anhui Agricultural University, Hefei, China. Nurses were collected when they were feeding the larvae inside cells. Foragers were collected when they were flying back to hive with pollen on their hind legs. Four typical colonies were used to provide regularly aged nurses (TC_N) and foragers (TC_F), with *N* = 30 for each group. One-day-old honey bees from the above four typical colonies were used to create four corresponding single-cohort colonies according to Liu et al. [57] and Ben-Shahar et al. [32]. In short, approximately 1000 one-day-old honey bees were kept in a small hive, which included an unrelated mated queen, an empty comb for the queen to lay eggs, and a comb containing some honey and pollen. In these single-cohort colonies, some honey bees (~ 5–10%) will differentiate into young foragers (7–9 days old, SCC_YF), while others will remain as normally aged (young) nurses (SCC_YN). We then removed the capped brood during the next 30 days, thereby forcing some nurses to continue nursing despite their old age (28–30 days old, SCC_ON), while foragers now were of similar ages (28–30 days old, SCC_OF) to those from typical colonies. We thus decoupled the behaviours of honey bee workers from their ages, which are linked in typical colonies. We sampled 30 bees each from these 4 types of bees from each of the 4 SCCs. All collected honey bees were immediately stored in liquid nitrogen for future RNA extraction.

### Library preparation for lncRNA sequencing

Total RNA was isolated from heads of each honey bee sample using TRIzol reagent (Invitrogen, USA). The quality and quantity of the RNA was assessed by the RNA Nano 6000 Assay Kit of the Bioanalyser 2100 system (Agilent Technologies, CA, USA). Three μg of RNA per honey bee sample was used as input material for RNA sample preparations. After removing the ribosomal RNA and rRNA-free residue, we used rRNA-depleted RNA to construct sequencing libraries using a NEBNext® Ultra™ Directional RNA Library Prep Kit for Illumina® (NEB, USA). M-MuLV Reverse Transcriptase, DNA Polymerase I and RNase H were used to obtained the first and the second strand cDNAs respectively. NEBNext Adaptor with a hairpin loop was ligated for hybridization. Finally, the products were purified using AMPure XP system (Beckman Coulter, Beverly, USA), and library quality was assessed with the Agilent Bioanalyser 2100 system. Our libraries were sequenced on an Illumina Hiseq 2500 platform.

### Data analysis

Clean reads were obtained by removing reads containing an adapter, reads containing ploy-N and low quality reads from raw data. QC calculation (Q20, Q30 and GC) were performed at the same time. Then clean reads with high quality were used for next analyses. Firstly, all clean reads were mapped to the reference genome (https://www.ncbi.nlm.nih.gov/assembly/GCF_000002195.4/) (http://biomirror.aarnet.edu.au/biomirror/ncbigenomes/Apis_mellifera/GFF//ref_Amel_4.5_top_level.gff3.gz). The index of honey bee genome (Amel 4.5) was built and paired-end clean reads were aligned to the genome. All mapped reads were assembled by both Scripture (beta2) [26] and Cufflinks (v2.1.1) [27]. Cufflinks included the assembler along with the utilities to structurally compare Cufflinks output between samples (Cuffcompare) and to perform differential expression testing (Cuffdiff). Cuffdiff was used to calculate fragments per kilo-base of exon per million fragments mapped (FPKMs) based on the length of the fragments, and the read counts mapped to this fragment for both lncRNAs and coding genes in each sample. Gene FPKMs were computed by summing the FPKMs of transcripts in each gene group. Cuffdiff provides statistical routines for determining differential expression in digital transcript or gene expression data using a model based on the negative binomial distribution. P-adjust< 0.05 and the absolute value of log_2_ (fold change) > 1 were set as the thresholds for significantly differentially expressed genes.

### Screening for provisional lncRNAs

**Fig. 6:**
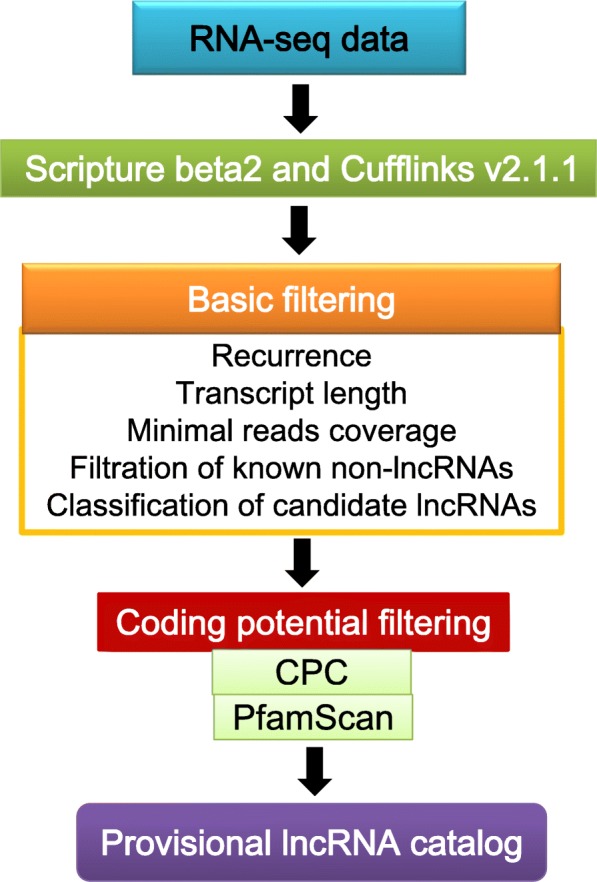
The main workflow for screening lncRNAs

The screening included basic filtering and coding potential filtering (Fig. 6). There were five steps of basic filtering: 1. recurrence: transcripts which were assembled by more than two software or detected in more than two samples were selected; 2. transcript length: transcripts with length of more than 200 bp were selected; 3. minimal read coverage: transcripts with FPKM of more than 2 were selected; 4. filtration of known non-lncRNAs; 5. classification of candidate lncRNAs: Coding Potential Calculator (CPC) and Pfam Scan (Pfam) were used to predict the coding potential. CPC (0.9-r2) was used to assess the protein-coding potential of a transcript based on biologically meaningful sequence features [58]. Pfam (v1.3) was used to identify the occurrence of any of the known protein family domains documented in the Pfam database [59]. Any transcript was excluded if it was predicted to possess coding potential by each of the two methods.

### Target gene prediction

In order to study the function of lncRNAs, we used *cis* and *trans* to predict the potential targets of lncRNAs. The C*is* role is the lncRNA acting on its neighbour genes. We searched protein-coding genes 10 k/100 k upstream and downstream of lncRNAs and then analysed their function. The *trans* role of lncRNAs was examined based on its expression correlation coefficient with protein coding genes (absolute value of Pearson correlation ≥ 0.95).

### GO and KEGG enrichment analyses

Gene Ontology (GO) enrichment analysis of differentially expressed mRNAs or target genes of lncRNAs was performed using the GOseq R package. Kyoto Encyclopedia of Genes and Genomes (KEGG) is a database resource for understanding high-level functions and effects of the biological system (http://www.genome.jp/kegg/). KOBAS (v2.0) was used to test the statistical enrichment of differential expression genes or target genes of lncRNAs in KEGG pathways [58].

### Quantitative PCR analysis

Total RNAs from the heads of honey bee from four typical and four single-cohort colonies were used for quantitative PCR analysis. Briefly, the first strand cDNA was obtained using a HiScript II Q RT SuperMix for qPCR (Vazyme, China) and were subjected to quantification of lncRNAs or mRNAs with *β-actin* as the housekeeping gene using a standard SYBR Green PCR kit (ChamQ™ SYBR Colour Qpcr Master Mix) (Vazyme, China) on the CFX Connect Real-Time System (Bio-Rad). The quantitative PCR was performed under the following conditions: 95 °C for 30 s, 40 cycles of 95.0 °C for 10 s and 60 °C for 30 s. Then, for melting curve analysis, temperatures were increased from 70 °C to 95 °C (at 0.5 °C increment every 5 s until plate reading). Each sample test was performed in triplicate for all reactions. Gene expression was quantified relative to the expression of *β-actin* using the comparative cycle threshold (ΔCT) method.

### Statistical analysis

All qPCR data were analysed using one-way analysis of variance (ANOVA) to test homogeneity of variances via Levene’s test and followed with Students’ T test (PASW Statistics 18.0 software). The results are shown as the mean ± standard error. *P* < 0.05 was considered to be statistically significant.

## Additional files

Additional file 1: Detailed information on six honey bee samples for RNA-seq. (XLS 25 kb)

Additional file 2: The distribution of reads mapped to reference annotation. (XLS 25 kb)

Additional file 3: A hierarchical heat map showing the transformed expression values for transcripts (mRNA and lncRNA). Red shows higher expression, and blue shows lower expression. (PPTX 347 kb)

Additional file 4: Common differentially expressed lncRNAs and mRNAs among the three comparisons. (XLS 134 kb)

Additional file 5: Venn diagram of common differential expression transcripts (lncRNA and mRNA) among two comparison groups (SCC_YF vs. SCC_YN and SCC_OF vs. SCC_ON). (PPTX 175 kb)

Additional file 6: The top 20 enriched pathways in the TC_F vs. TC_N (A), SCC_YF vs. SCC_YN (B) and SCC_OF vs. SCC_ ON (C) comparisons (*cis*). (XLSX 12 kb)

Additional file 7: The significantly enriched GO terms (corrected *p*-value < 0.05) detected in the TC_F vs. TC_N, SCC_YF vs. SCC_YN and SCC_OF vs. SCC_ON comparisons (*trans*). (XLS 68 kb)

Additional file 8: The top 20 enriched KEGG pathways in the TC_F vs. TC_N, SCC_YF vs. SCC_YN and SCC_OF vs. SCC_ ON comparisons (*trans*). (XLS 30 kb)

Additional file 9: The significantly enriched GO terms (corrected *p*-value < 0.05) of differentially expressed mRNAs from the three comparison groups. (XLS 74 kb)

Additional file 10: The top 20 enriched KEGG pathways of differentially expressed mRNAs from the three comparison groups. (XLS 31 kb)

## Abbreviations

ACS: Acyl-CoA synthetases
ACSF2: acyl-CoA synthetase family member 2
cGMP: cyclic guanosine monophosphate
CPC: Coding Potential Calculator
DAMB: dopamine receptor in mushroom bodies
DNTPs: deoxy-ribonucleotide triphosphate
dop1: dopamine receptor, D1
dTTP: deoxy- thymidine triphosphate
dUTP: deoxyuridine triphosphate
HR38: hormone receptor-like in 38
JH: juvenile hormone
*Kr-h1*: *Krüppel-homolog 1*
mrjps: major royal jelly proteins
PFAM: Pfam-scan
Pkc: protein kinase C
qPCR: quantitative PCR
Vg: vitellogenin
