# Supplementary figures and images for: lncRNA profile of Apis mellifera and its possible role in behavioural transition from nurses to foragers
Source: BMC Genomics. 2019 May 21;20:393. doi: 10.1186/s12864-019-5664-7 (PMC6528240; doi:10.1186/s12864-019-5664-7)

## Slide 1
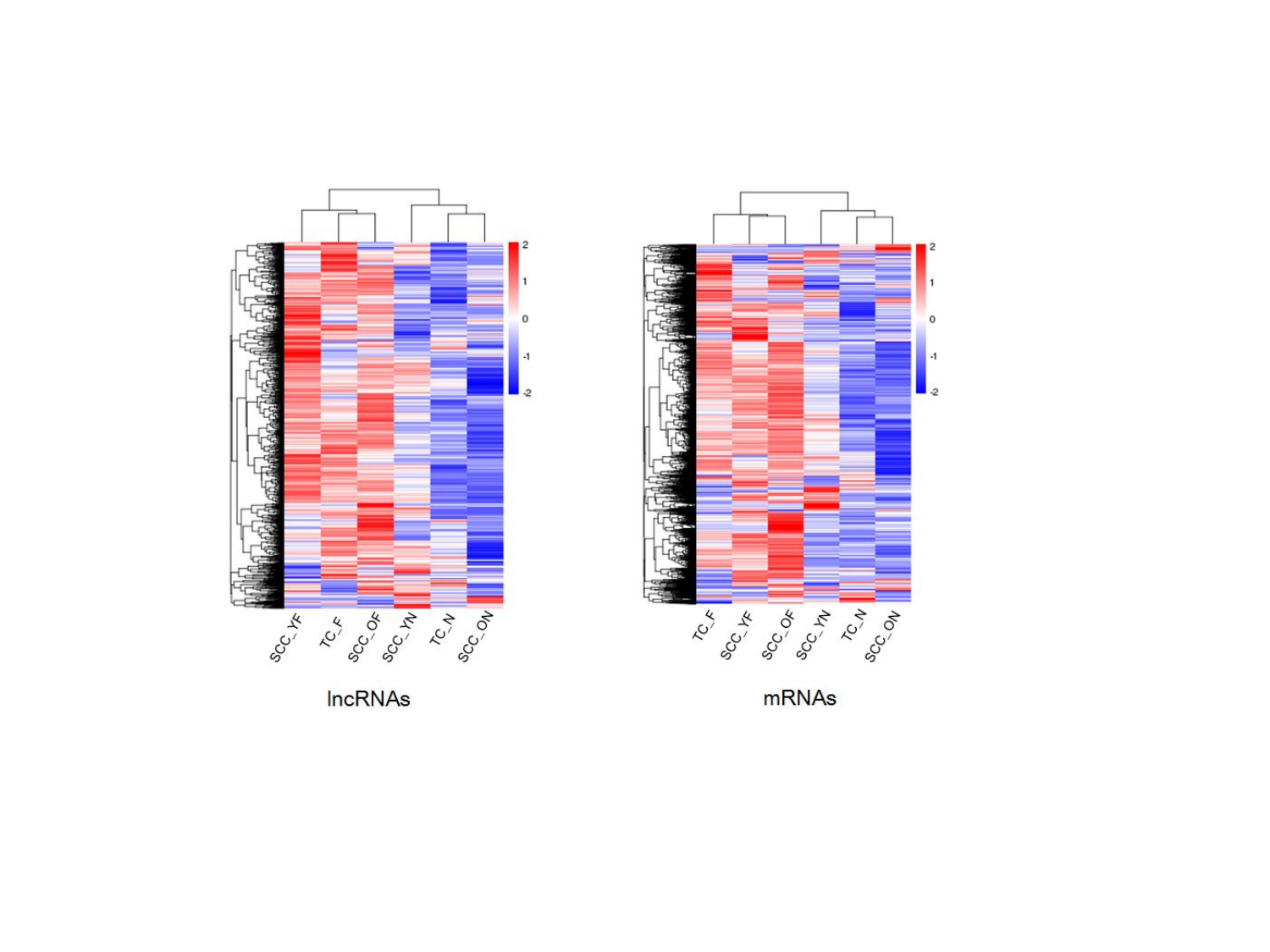

Supplement: Supplementary file 3 — A hierarchical heat map showing the transformed expression values for transcripts (mRNA and lncRNA). Red shows higher expression, and blue shows lower expression. (PPTX 347 kb) [file 12864_2019_5664_MOESM3_ESM.pptx]

## Slide 1
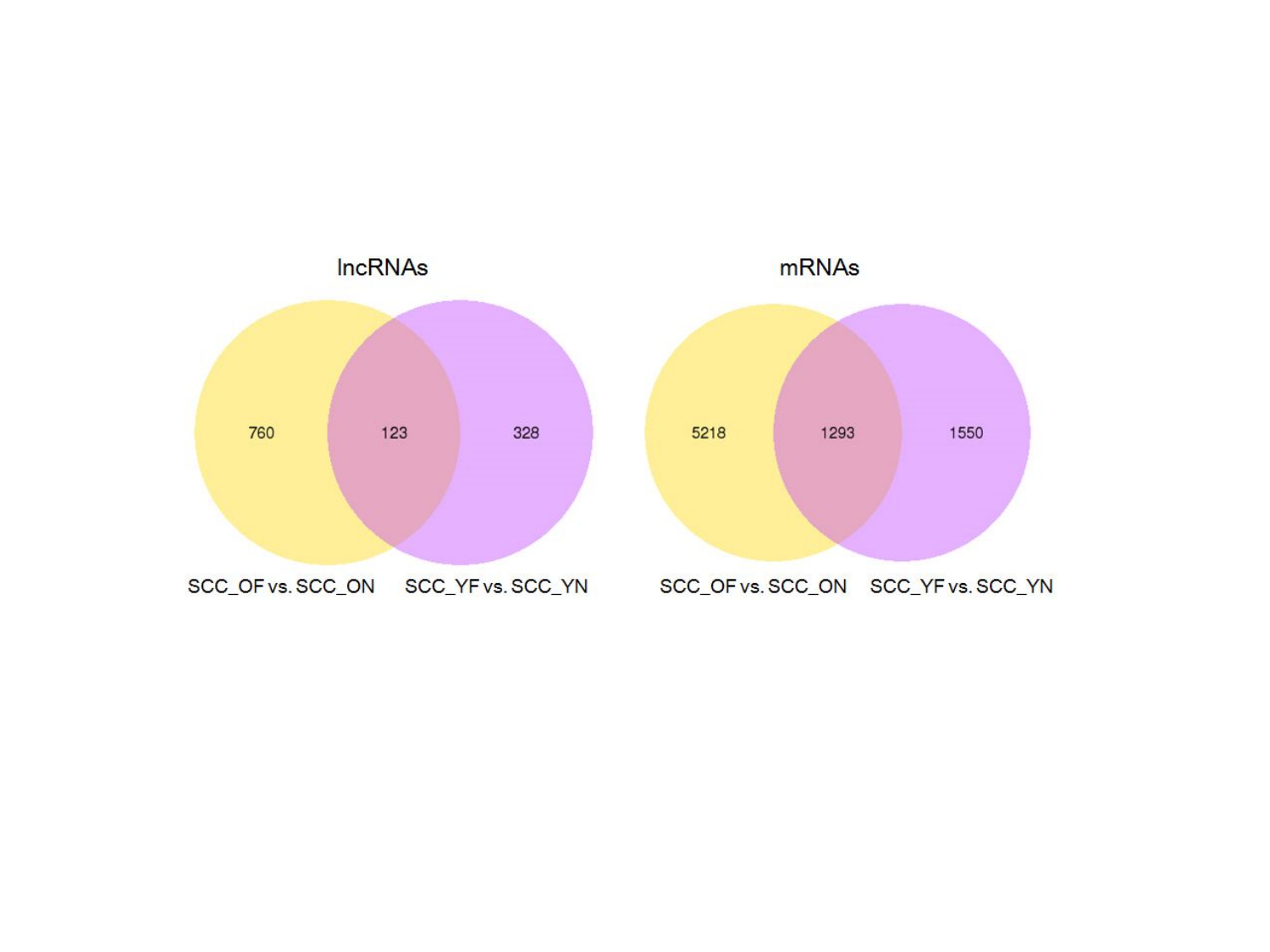

Supplement: Supplementary file 5 — Venn diagram of common differential expression transcripts (lncRNA and mRNA) among two comparison groups (SCC_YF vs. SCC_YN and SCC_OF vs. SCC_ON). (PPTX 175 kb) [file 12864_2019_5664_MOESM5_ESM.pptx]
